# Dual-mode imaging and therapeutic effects of drug-loaded phase-transition nanoparticles combined with near-infrared laser and low-intensity ultrasound on ovarian cancer

**DOI:** 10.1080/10717544.2018.1507062

**Published:** 2018-10-20

**Authors:** Shuning Chen, Yujiao Liu, Shenyin Zhu, Chunyan Chen, Wan Xie, Linlin Xiao, Yi Zhu, Lan Hao, Zhigang Wang, Jiangchuan Sun, Shufang Chang

**Affiliations:** a Department of Obstetrics and Gynecology, The Second Affiliated Hospital of Chongqing Medical University, Chongqing, PR China;; b Institute of Ultrasound Imaging, Second Affiliated Hospital of Chongqing Medical University, Chongqing, PR China;; c Department of Pharmacy, The First Affiliated Hospital of Chongqing Medical University, Chongqing, PR China

**Keywords:** Paclitaxel, indocyanine green, phase-shift nanoparticles, hypoxia, dual-mode imagin

## Abstract

Chemotherapy and photo-sonodynamic therapy (PSDT) can be combined through drug delivery nano-platforms to enhance the anti-tumor efficacy, however, which is limited by hypoxia in tumor, thereby causing chemotherapy resistance. Perfluoropentane (PFP) has the ability to carry oxygen and to enhance ultrasound or photoacoustic imaging after vaporization. Herein, we constructed a kind of nanoparticles (PTX/ICG and oxygen loaded PLGA nanoparticles (PIO_NPs)), which had PFP core carrying oxygen and PLGA shell loaded indocyanine green (ICG) and paclitaxel (PTX). PIO_NPs harbored good optical stability and the ability to transit phase. Moreover, it could rapidly release PTX and generate ROS under the mediation by near-infrared laser and low-intensity ultrasound. The PIO_NPs enhanced contrast of the ultrasound and PA imaging. In particular, PIO_NPs may be used to monitor and guide treatment for the accumulation of PIO_NPs at tumor site can be observed by PA imaging. Compared with PTX or other nanoparticles, PIO_NPs combined with laser and ultrasound (L.U) significantly induced apoptosis of SKOV3 cells and inhibited SKOV3 tumor growth. Therefore, PIO_NPs are of great potential in cancer imaging and therapy.

## 1. Introduction

Ovarian cancer is one of the most prevalent gynecologic malignancies with high incidence and mortality rates (Torre et al., 2015; Siegel et al., 2017). The standard treatment for ovarian cancer encompasses maximal cytoreductive surgical debulking followed by administration of paclitaxel (PTX )-platinum based chemotherapy. However, lack of early diagnosis and chemotherapy resistance tend to contribute to the high recurrence rate and poor prognosis of patients (Tew et al., 2014; Kampan et al., 2015; Cortez et al., 2018).

Combination therapy, combining two or more therapies together, has a great potential for improving therapeutic efficiency and overcoming drug resistance. Previous studies have explored the combination of photodynamic therapy (PDT) or sonodynamical therapy (SDT) and chemotherapy (Zheng et al., 2013; Huang et al., 2014; Su et al., 2015). Photo-sonodynamic therapy (PSDT) is a novel modality for cancer treatment, aimed at enhancing anticancer effects by the combination of PDT and SDT. By activating con-generous sensitizers with light and sound, PDST produced more obvious anti-cancer effects than any monotherapy, and further decreased the dosage of sensitizer and the energy of ultrasound or light which can further reduce the side effects (Wang et al., 2009; Tserkovsky et al., 2012; Li, et al., 2013, Wang et al., 2015; Liu et al., 2016a; Tang et al., 2017). Therefore, PSDT was used in our study as a more effective treatment. In spite of the complicated mechanism of PDST, the production of reactive oxygen species (ROS) is one of the key factors. Intracellular ROS production threatens the integrity of various biomolecules (Tomankova et al., 2009), leading to cellular damage and dysfunction (Kim et al., 2013; Di Meo et al., 2016). However, the effect of combination of PSDT and chemotherapy is rarely explored.

PTX is one of the main drugs for ovarian cancer chemotherapy (Kampan et al., 2015). Due to its high insolubility in water and severe anaphylactic reactions, its clinical applications are limited (Raisch et al., 2011). Indocyanine green (ICG) is a proven con-generous sensitizer of PSDT that responds to near-infrared light and ultrasound (Nomikou et al., 2012; Tang et al., 2017). Due to its low toxicity, ICG has been approved by the FDA for clinical application. However, the fast body clearance (plasmatic half-time of 2–4 min) and instability in water solution are considered as the main drawbacks of using ICG (Polom et al., 2011; Porcu et al., 2016). Liquid perfluorocarbon (PFC) compounds are good oxygen carriers (Riess, 2006) and can be vaporized using acoustic or optical droplet vaporization methods (Krafft, 2001; Sun et al., 2014). To improve the therapeutic effect, we consider the combination of PTX, ICG, and PFC through the drug delivery platform. And poly (lactic-co-glycolic acid) (PLGA) is a good choice for a drug delivery vehicle because of its biocompatibility and safety. Moreover, several studies have demonstrated that dyes-loaded or drug-loaded PLGA nanoparticles can induce PDT/SDT or enhance cytotoxicity (McEwan et al., 2014; Heo et al., 2015; Pakulska et al., 2016; Abou-ElNaga et al., 2017). Herein, our ideal drug delivery platform is based on PLGA, which has the following advantages: (1) high drug loading efficiency; (2) PTX-based chemotherapy combined with ICG-based PDST; (3) oxygen carried PFC improve hypoxia of tumor (McEwan et al., 2015; McEwan et al., 2016); (4) controlled drug release.

Due to the moderate fluorescence quantum yield of ICG (Porcu et al., 2016) and its absorption of NIR range, it has been suggested as a contrast agent in photoacoustic (PA) imaging. As a noninvasive imaging technique that converts optical signals into sound signals, PA imaging has great potential for disease diagnostic and therapeutic monitoring (Valluru et al., 2016). Various photosensitizer nano-composites have been used for PA imaging (Cho et al., 2010; Liu et al., 2016b; Zhang et al., 2016; Lemaster & Jokerst, 2017). Among them, ICG-based nanoparticles can be used as PA contrast agents due to their intense and stable signal (Chen et al., 2016; Gao et al., 2016; Deng et al., 2017). After being vaporized *via* optical irradiation and acoustic pressure, PFC can change its phase and subsequently generate gas (Krafft, 2001). And based its phase-shift ability, some studies synthesized PFC-loaded particles to enhance ultrasound images (Lin et al., 2017) and PA images (Wilson et al., 2012). Furthermore, a photosensitizer and PFC loaded particle can enhance dual mode imaging which combines PA and ultrasound imaging (Hannah et al., 2014; Chen et al., 2016; Lin, et al., 2017).

In this study, we constructed a nanoparticle with an oxygen-containing perfluoropentane (PFP) liquid core and PTX/ICG loaded PLGA shell. On the one hand, it can remarkably promote anti-tumor effects and effectively reduce drug resistance. Exposure to NIR and ultrasound contributed to controlled release of PTX and generation of ROS, localized treatment, and minimized side effects. On the other hand, based on the optical properties of ICG and the phase-shift capability of PFP, the nanoparticle can be a promising tool for dual mode imaging. This nanoparticle harbored a potential to integrate imaging diagnostics and anti-tumor therapy and to realize medical imaging-guided visual diagnosis and treatment.

## 2. Materials and methods

### 2.1. Materials

PTX was purchased from Nantong Feiyu Biotechnology (Jiangsu, PR China). ICG was purchased from Aladdin (Shanghai, PR China). PLGA (lactide: glycolide = 50:50, molecular weight = 12,000 Da) was obtained from the Shandong Key Laboratory of Medical Polymer Materials (Shangdong, PR China). Perfluoro-*n*-pentane (PFP) was obtained from Fluka (St Louis, MO). Polyvinyl alcohol (PVA, 87–90%) was purchased from Sigma-Aldrich Co. (St Louis, MO). Singlet oxygen sensor green (SOSG) was obtained from Thermo Fisher Scientific (Waltham, MA). 3-(4,5-dimethylthiazol-2-yl)-2,5-diphenyltetrazoliumbromide (MTT), 2-(4-amidinophenyl)-6-indolecarbamidine dihydrochloride (DAPI) and 2’,7’-dichlorofluorescin diacetate (DCFH-DA) were all purchased from Beyotime Biotechnology (Shanghai, PR China). All other reagents were commercial products of analytical grade.

### 2.2. Methods

#### 2.2.1. Preparation of particles

PTX/ICG and oxygen loaded PLGA nanoparticles (PIO_NPs) were prepared using a modified double emulsion (water/oil/water) evaporation process according to previous studies (Tang et al., 2017). Saturated ICG aqueous solution and 200 μL PFP bubbled with oxygen gas were mixed using an ultrasonic probe (Sonics & Materials, Inc., Fairfield, CT) for 1 min. The mixture was bubbled with oxygen again. Next, the mixture was added to methylene chloride (2 mL) dissolving PLGA (50 mg) and PTX (3 mg) and emulsified using ultrasonic probe at 120 W for 3 min. Then, the above-described emulsified solution was poured into PVA solution (4% w/v) and sonicated again to produce second emulsion. The final emulsion was stirred for 4 h to sufficiently extract methylene chloride. Subsequently, the solution was centrifuged at 12,000 rpm for 5 min at 4 °C (Biofuge Stratos Centrifuge; Thermo Fisher Scientific, Germany) , followed by washing by deionized water of the precipitate. The process of centrifugation was repeated three times until the supernatant was clarified. The washed precipitate was re-suspended in 5 mL oxygen-enriched PBS and stored at 4 °C for further use. The IO_NPs (ICG and oxygen loaded PLGA nanoparticles) were prepared without PTX similarly. The O_NPs (only oxygen loaded nanoparticles) were prepared without ICG and PTX. PI_NPs (PTX and ICG loaded PLGA nanoparticles) were prepared omitting the oxygen saturation processes. All procedures were carried out under low temperature and dim light.

#### 2.2.2. Characterization of the NPs

The NPs size (diameter, nm), polydispersity index and surface charge (zeta potential, mV) were measured by dynamic light scattering using a Malvern Zetasizer Nano ZS unit (Malvern Instruments, Malvern, UK) at room temperature. In addition, these indicators of PIO_NPs were determined after laser irradiation (808 nm, 1.5W/cm^2^ for 2 min). The morphology of PIO_NPs with or without laser irradiation was obtained by microscopy (Eclipse Ti, Nikon Corporation, Tokyo, Japan). The morphology of PIO_NPs was also detected by scanning electron microscope (SEM, FEI Inspect F50, FEI Company, USA), and transmission electron microscope (TEM, FEI Tecnai G2 F20, FEI Company, USA).

The LE and EE of PTX were detected by HPLC analysis. A C18-column was used with a mobile phase composed of acetonitrile and water (70: 30, v/v) and flow rate of 1.0 mL/min. The LE and EE of ICG were determined in triplicate by an ultraviolet-visible (UV–Vis) spectrophotometer (260-Bio, Thermo Fisher Scientific). The absorbance of non-entrapped ICG in the supernatant was measured at 780 nm. The mass of entrapped ICG was equal to mass of total ICG minus the mass of non-entrapped. The LE and EE were calculated according to the following formulas: LE (%) = (weight of loaded drug/total weight of NPs) × 100, EE (%) = (weight of loaded drug/weight of initially added drug) × 100.

The absorption spectra and the fluorescence spectra of free ICG and PIO_NPs were detected in PBS and 10% fetal bovine serum by similar procedure in previous studies (Zheng et al., 2011; Ma et al., 2017). The absorption spectra were determined by UV–Vis spectrophotometer (wavelength from 650 to 850 nm). The fluorescence spectra were obtained by fluorescence spectrometer (Cray Eclipse, Agilent Technologies) with excitation at 740 nm, meanwhile, the emission spectra were recorded from 760 to 860 nm. Furthermore, to monitor the degradation of ICG in different samples, the absorbance (at 780 nm) and the fluorescence intensity (at maximal emission wavelength) of samples were recorded every 3 d for 15 d.

*In vitro* PTX release profile of PIO_NPs was also determined by HPLC. The 1 mL PIO_NPs was placed into vial containing 50 mL of phosphate buffered saline (PBS, pH 7.4). The vials, containing equal PIO_NPs, were shaken continuously for 48 h at 120 rpm and 37 °C. Then samples were ultra-centrifuged and collected at the indicated time points (0, 2, 4, 8, 12, 24, 36, and 48 h). We also measured PTX release behavior after NIR irradiation (808 nm, 1.5W/cm^2^ for 2 min) and ultrasound exposure (1.0 MHz, 1W/cm^2^, 1 min). The PTX content was measured using the same HPLC method as above.

The SOSG reagent was highly selective for ^1^O_2_. In the presence of singlet oxygen, it emitted green fluorescence (excitation/emission maxima ∼504/525 nm). The SOSG and free ICG, O_NPs, PI_NPs, or PIO-NPs were mixed into MilliQ water to obtain working concentrations of 5 μM SOSG and 5 μg/mL ICG in final solution. Next, the solution was irradiated with 1.5W/cm^2^ 808 nm for 2 min and exposed to 1.0 W/cm^2^ low-intensity for 1 min (The role of laser and ultrasound [L.U]). Finally, singlet oxygen, before and after explosion to L.U, was determined by measuring the fluorescence intensity using a fluorescence microplate reader (Varioskan Flash, Thermo Fisher Scientific).

#### 2.2.3. Therapeutic effect *in vitro*

The human ovarian cancer SKOV3 cell line was kindly offered by Professor Ronald X. Xu at University of Science and Technology of China (Hefei, Anhui). Cells were maintained in RMPI 1640 culture medium containing 10% fetal bovine serum, 1% penicillin, and 1% streptomycin at 37 °C under 5% CO_2_. Cells in the exponential phase of growth were used for all experiments.

SKOV3 cells (1 × 10^6^ cells/well) were seeded into a six-well plate and incubated overnight. Then, the medium was replaced by the free medium containing ICG (1.73 μg/mL) or PIO_NPs (containing ICG 1.73 μg/mL, PTX 3.64 μg/mL). After 4 h incubation, the cells were washed thrice with PBS and fixed with 4% paraformaldehyde for 15 min. The nuclear dye DAPI was used as a positive control to stain nuclei in the experiment. Finally, the cells were observed under confocal laser scanning microscope (CLSM, Leica TCS SP8, Heidelberg, Germany). Similarly, SKOV3 cells were treated and subjected to flow cytometry.

To determine the role of apoptosis in response to different NPs combined with L.U, we assessed the apoptosis of cells after treatment using Annexin V-fluorescein isothiocyanate (FITC)/PI double staining, followed by analysis of flow cytometer. Briefly, the SKOV3 cells were seed into six-well plate in 1 mL of medium and cultured for 24 h. Next, the medium was removed and replaced by 250 μL culture media containing equivalent concentrations of free PTX, IO_NPs, PI_NPs, and PIO_NPs (final ICG concentration 3.46 μg/mL, PTX 7.28 μg/mL). After 4 h incubation, sterile PBS was used to wash cells thrice and each well was added with new complete medium. Then the treated cells were irradiated with 1.5W/cm^2^ 808 nm for 2 min and exposed to 1.0 W/cm^2^ low-intensity for 1 min (The role of L.U). Cells were collected after 24 h of treatment and re-suspended with staining solution containing V-FITC and PI. All samples were incubated at room temperature in dark for 15 min and analyzed by flow cytometer.

Intracellular ROS productions were determined by using 2’,7’-dichlorofluorescin diacetate (DCFH-DA) assay. Briefly, SKOV3 cells were seed in six-well plate (1 × 10^6^ cells/well) and incubated overnight. Next, cells were incubated with PTX, IO_NPs, PI_NPs, or PIO_NPs for 4 h. The cells were washed three times by using PBS and incubated by DCFH-DA (10 μM) in dark for 30 min. Unloaded DCFH-DA was discarded and replaced by medium. The test samples were exposed to L.U, followed by observation by fluorescence microscope. After interacted with intracellular ROS, DCFH changed to DCF with green fluorescence. In addition, fluorescence microplate reader was employed to determine the fluorescence intensity at excitation of 485 nm and emission of 528 nm.

#### 2.2.4. Imaging *in vivo*

Some similar tumor-bearing nude mice were anesthetized with 1% sodium pentobarbital. Then, B-mode and PA-mode imaging were performed using VEVO LASR PA imaging system (VIVO 2100, FUJIFILM Visual Sonic, Inc., Bothell, WA). The mice were divided into 3 groups (*n* = 3), which were injected with PBS, free ICG or PIO_NPs through tail vein. Images were obtained at 2, 4, 6, 12, 14, 48, and 72 h, respectively. In addition, in order to explore the changes in the imaging after laser irradiation, the tumors were subjected to NIR irradiation (1.5W/cm^2^, 5 min) at the best imaging time (6 h), followed by analysis of the echo intensity (EI) in B-Mode using DFY (invented by the Institution of Ultrasound Imaging of Chongqing Medical University) and the PA average value using the VEVO LASR PA imaging system (VIVO 2100, FUJIFILM Visual Sonic, Inc., Bothell, WA).

#### 2.2.5. Therapeutic effect *in vivo*

Female BLAB/c athymic nude mice, 6–7 weeks of age and 18–20 g of weight, were purchased from Chinese Academy of Medical Science (Beijing, China). SKOV3 cells in logarithmic growth phase were trypsinized, washed twice, and suspended in sterilized PBS. The 200 μL cell suspension (5 × 10^7^ cells/mL) was injected into flank of each mouse. The experiments were all conducted in compliance with Practice Guidelines for Laboratory Animals of China.

Ten days after tumor growth, the tumor volume reached about 500 mm^3^. The following formula was used to estimate tumor volume: length × width^2^ × 0.5. Then these mice were randomly divided into five groups (eight mice per group) and injected different drugs of 300 μL through tail vein. Specifically, the treatment groups were as follows: (1) control (saline); (2) PTX (only PTX); (3) PI_NPs + L.U (applying PI_NPs combined L.U); (4) IO_NPs + L.U (applying IO_NPs combined L.U); (5) PIO_NPs + L.U (applying PIO_NPs combined L.U). The PTX dose (5 mg/kg) was kept consistent in these above treatment groups. For groups (3), (4), and (5), 808 nm laser (1.5W/cm^2^, 5 min) and low-intensity ultrasound (1W/cm^2^, 1 min) were applied 6 h after injection. Treatment was performed every 5 d for a total of four times. Body weight and tumor volume were recorded every three days throughout the treatment circle.

Twenty-four hours after the last treatment, three mice were sacrificed randomly in each group. Their tumor tissues and organs (heart, liver, spleen, lung, and kidney) were harvested and fixed in 4% paraformaldehyde for paraffin clines. Hematoxylin and eosin (H&E) staining was used for histopathological analysis. Terminal-deoxynucleotidyl Transferase Mediated Nick End Labeling (TUNEL) assay was performed to detect apoptosis in tumor cells, and apoptotic index (AI) was used to show the results. The tumor tissue sections were stained with antibodies against CD34 (polyclonal, 1:1000, Abcam, Cambridge, UK) and VEGF (polyclonal, 1:200, Affinity, Sterling, VA) to detect the microvascular density. The result of VEGF was expressed using IOD integrated optical density (IOD). And the microvessel density (MVD) was determined by calculating all CD34 positive vessels. The pictures were obtained by a digital camera and analyzed by software Image Pro Plus version 6.0 (Media Cybernetics, Inc., Rockville, MD, USA) .

Western blot was used to detect the expression of HIF-1α and MDR-1 in tumor tissues. Additionally, the remaining five mice in each group were observed until natural death, and the survival time of each mouse was recorded.

### 2.3. Statistical analysis

Graphpad Prism version 6.0 (La Jolla, CA) software was used to analyze the experimental results. The data were expressed as mean ± standard deviation (SD) and analyzed by one-way analysis of variance (ANOVA). The differences among the means in *in vivo* experiments were evaluated by the Tukey Kramer multiple comparison test. Survival rates of different treatment groups were illustrated by the Kaplan–Meier curves and compared by the Log-rank Test. A *P* value of less than .05 was considered as statistically significant.

## 3. Results and discussion

### 3.1. Characterization of the NPs

**Scheme 1.**
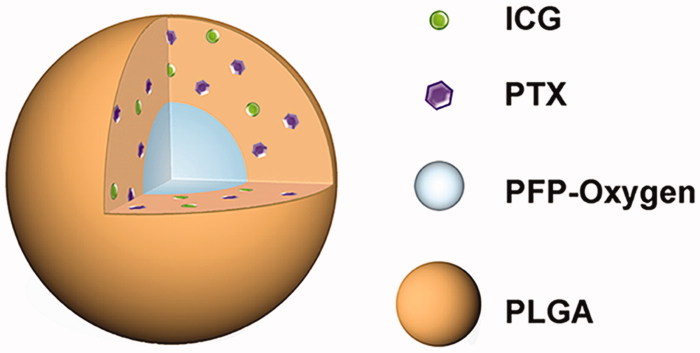
Schematics illustration of the PIO_NPs. PLGA: poly (DL-lactide-co-glycoclic acid); PFP-oxygen: perfluoro-n-pentane carried oxygen; PTX: paclitaxel; ICG: indocyanine green; PIO_NPs: paclitaxel, indocyanine green and oxygen loaded nanoparticles.

**Figure 1.**
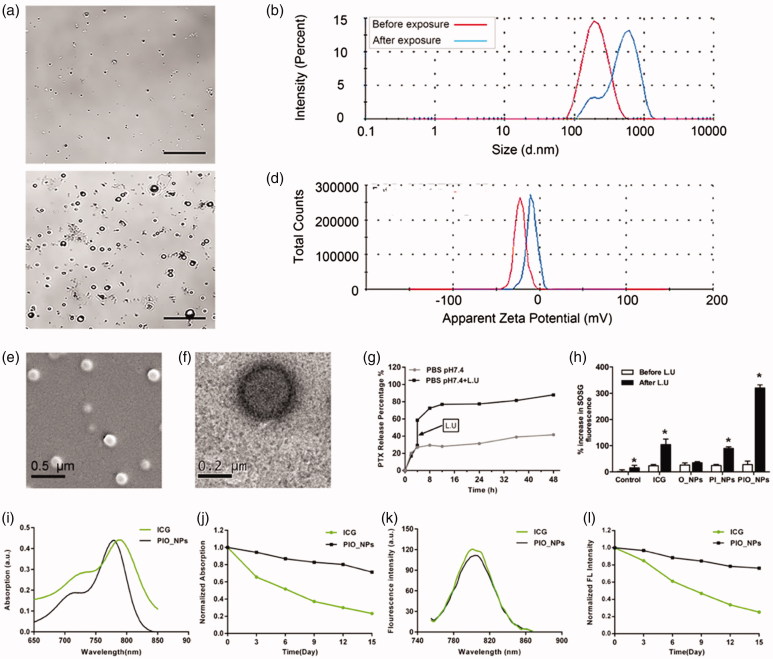
Optical microscopy images before (a) and after (b) laser irradiation, scale bar of microscopy images are 10 μm. Size distributions (c) and zeta potential (d) were detected before (red line in pictures) and after (blue line in pictures) laser irradiation. SEM (e**)** images of PIO_NPs, the scale bar is 0.5 μm. TEM (f) images of PIO_NPs, the scale bar is 0.2 μm. The release of PTX with or without L.U exposure (g), percentage increase of SOSG fluorescence intensities for control, free ICG, O_NPs, and PIO_NPs in MilliQ water with or without L.U exposure (h). The role of laser and ultrasound (L.U). Error bars represented ± the standard error where *n* = 3. *p < .05 versus each group without L.U. Absorbance spectra of free ICG and PIO_NPs (i), absorbance stability test of free ICG and PIO_NPs (j), fluorescence spectra of free ICG and PIO_NPs (k), and fluorescence stability test of free ICG and PIO_NPs (l).

The schematic structure of PIO_NPs was shown in Scheme 1. The PIO_NPs contained a liquid oxygen-saturated PFP core and the PLGA shell loaded ICG and PTX. And the optical microscopy image, size distribution, and zeta potential of PIO_NPs were acquired (Figure 1(a), and red line in Figure 1(b,d)). The phase change was then performed by 808 nm laser irradiation, and the nanoparticles were measured again (Figure 1(c), and blue line in Figure 1(b,d)). After PIO_NPs triggered by laser irradiation to phase transition, its size was significantly increased and the zeta potential was slightly increased. SEM image and TEM image of PIO_NPs are shown in Figure 1(e,f). The results showed that PIO_NPs were generally spherical in shape with good monodispersity. As shown in Table S1, the average diameter of PIO_NPs was 186.4 ± 1.9 nm; and the average zeta potential was −19.43 ± 0.55 mV. The drug encapsulation efficiency (EE) and drug loading efficiency (LE) of PTX or ICG were also shown. For PIO_NPs, the EE and LE of PTX were 64.29 ± 2.76 and 3.64 ± 0.16%, the EE and LE of ICG were 57.27 ± 0.77 and 1.73 ± 0.15%. As an ideal phase-changeable drug-loaded nanoparticle, PIO_NPs not only encapsulate PTX and ICG, but also can be triggered to phase transition by laser irradiation. These properties of other nanoparticles including IO_NPs and PI_NPs were compared with PIO_NPs (Table S1), and the LE and EE of ICG or PTX were lower than those of PIO_NPs.

The optical properties of PIO_NPs were determined. The absorption spectrum of free ICG and PIO_NPs was shown in Figure 1(i), while the fluorescence spectra were shown in Figure 1(k). Although the ICG entrapped into PLGA (PIO_NPs) exhibited a near-infrared absorption with absorbance peak at 790 nm, the absorption peak was hypochromic-shifted 10 nm approximately. On the other hand, the emission peak of PIO_NPs was changed almost 6 nm whose peak was red-shifted from 788 to 804 nm. We also investigated the optical stability of free ICG and PIO_NPs, measured the absorption and emission spectra separately every 3 d, which revealed that free ICG was degraded faster than PIO_NPs. After 15-d observation, PIO_NPs decreased the intensity of absorption by about 25%, while free ICG was decreased nearly 80% (Figure 1(j)). In addition, compared with free ICG, PIO_NPs have better absorption stability in 10% fetal bovine serum. (Figure S2). The fluorescence intensity of PIO_NPs was decreased by about 20%, but free ICG was decreased by nearly 80% (Figure 1(l)). After the ICG loaded into the nanoparticles, its absorption and fluorescence stability were significantly improved, which was favorable for the long-term storage.

To further investigate the potential of PIO_NPs for chemotherapy and PSDT, we measured the release of PTX and the production of singlet oxygen after L.U exposure. At 37 °C and pH 7.4, only 41.65% PTX released after 48 h. However, the release of PTX was increased from 29.04 to 58.58% with L.U exposure at 4 h, finally reaching 86.65% at 48 h (Figure 1(g), Figure S1), which indicated that PTX release was faster when mediated by L.U. SOSG was used for detecting production of singlet oxygen based on its fluorescence changing. A plot of percentage increased in SOSG fluorescence before and after L.U treatment for PBS, free ICG, O_NPs, PI_NPs, and PIO_NPs was shown in Figure 1(h). After exposure, the SOSG fluorescence intensity revealed a significant increase for PIO_NPs (319.76%) when compared with free ICG (104.1%), O_NPs (35.21%), or PI_NPs (89.99%). It suggested that the production of singlet oxygen was not only due to the excitation of the ICG, but also depended on the supply from PFP carried oxygen.

### 3.2. Therapeutic effect *in vitro*

**Figure 2.**
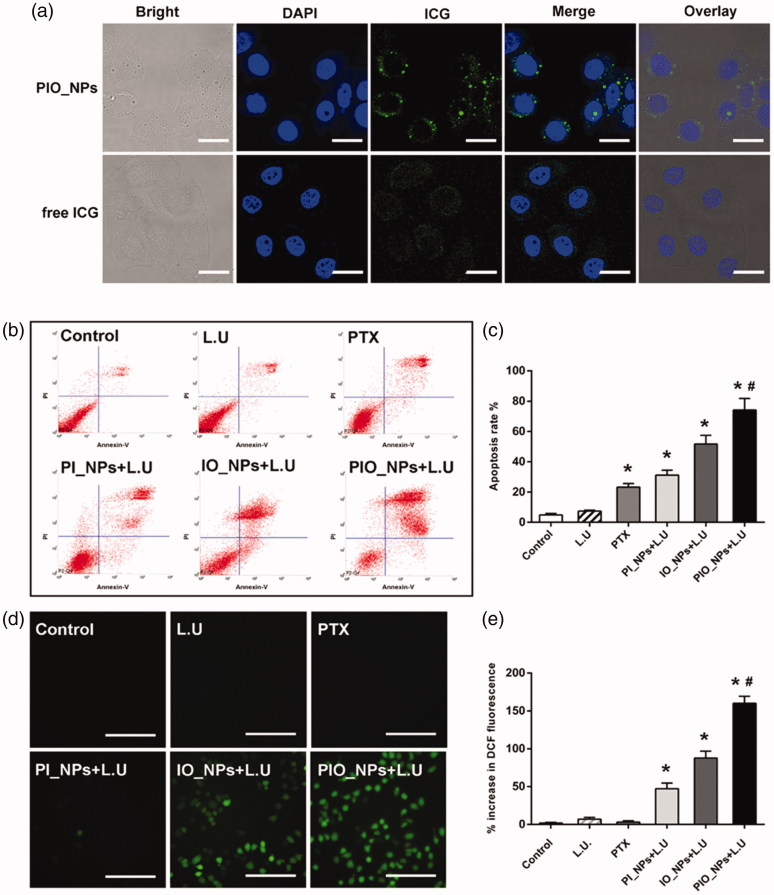
Cellular uptake of free ICG and PIO_NPs by SKOV3 cells (a), scale bar is 20 μm. Cell apoptosis in SKOV3 cells with different treatment (b). The percentage of apoptosis cells was determined by flow cytometry 24 h after treatment (c). Data were represented as mean ± SD (*n* = 3). Each treated group compared with control group, *p < .05; PIO_NPs + L.U group compared with other groups, #*p* < .05. SKOV3 cell incubated with DCFH-DA staining for ROS detection by fluorescence microscopy images (d), scale bar is 50 μm. Percentage increase of DCF fluorescence of distinct groups was (e). Each treated group compared with control group, *p < .05; PIO_NPs + L.U group compared with other groups, #*p* < .05.

The cell uptake behavior of free ICG or PIO_NPs was observed by confocal laser scanning microscope. As shown in Figure 2(a), cells incubated with PIO_NPs exhibited stronger intracellular green fluorescence signal. Due to instability of ICG, cells incubated with free ICG exhibited weak intracellular green fluorescence signal. In addition, it is possible to remove part of the ICG in the medium by washing three times with PBS. For the PIO_NPs group, a large number of PIO_NPs had been up-taken by the cells. And in bright image, there were many visible particles in cytoplasm. Further quantitative analysis by flow cytometry (Figure S3) revealed a significant increase of fluorescence intensity in PIO_NPs group. The mean fluorescence intensity of the PIO_NPs group was about 15 times that of the ICG group. These results demonstrated that PIO_NPs can be uptake into SKOV3 cells and also keep its fluorescence characteristic.

The cell viability was detected by MTT assay after different treatment for 24 h. Compared with PTX, PIO_NPs containing the same concentration of PTX reduced cell viability to a great extent (Figure S4(a)). This may be due to that PIO_NPs were effectively uptake by cells while PTX was slowly released. After exposure to L.U, the cell viability was further reduced in PIO_NPs group. Combined with L.U, PIO_NPs were more efficient for treatment. We also assessed the cytotoxicity of different treatment (Figure S4(b)). As a result, the cell viability of PIO_NPs + L.U group was 38.17 ± 4.41%, which was almost a half of PTX group. Cell viability of the PI_NPs + L.U group and the IO_NPs + L.U group was 69.96 ± 3.10% and 59.42 ± 2.78%, respectively, indicating that NPs without PTX loaded or oxygen carried exerted less effect on cell viability. In addition, it was shown that only exposure to L.U had no significant killing effect on the cells. The rate of apoptosis was quantified by flow cytometry (Figure 2(b,c)). The apoptotic trend was consistent with the cell inhibition rate in MTT assay for each group. To be specific, the apoptotic rate in the PIO_NPs group reached 74.09 ± 7.64%, while that the other groups did not exceed 50%. These results demonstrated that PIO_NPs as a drug delivery system can combine the chemotherapeutic effects of PTX with the photo-sono dynamics of ICG.

Generation of ROS, as a primary factor to induce cell apoptosis in photo-sono dynamic treatment (Krafft, 2001; Riess, 2006; Polom et al., 2011; Porcu et al., 2016), was also detected (Figure 2(d,e)). After interacting with intracellular ROS, DCFH changed to DCF with green fluorescence. Cells treated with PTX or only L.U hardly produced intracellular ROS. As confirmed by previous experiments, exposure of ICG-treated cells to NIR or ultrasound resulted in the production of ROS (Zheng et al., 2013). The NPs containing ICG (PI_NPs, IO_NPs, and PIO_NPs) significantly increased intracellular DCF fluorescence under L.U. In PIO_NPs + L.U group, the DCF fluorescence increased by 160.04 ± 9.20%, higher than other NPs groups. This may be due to the oxygen it carried, which could be effectively triggered by NIR and ultrasound.

### 3.3. Imaging *in vivo*

**Figure 3.**
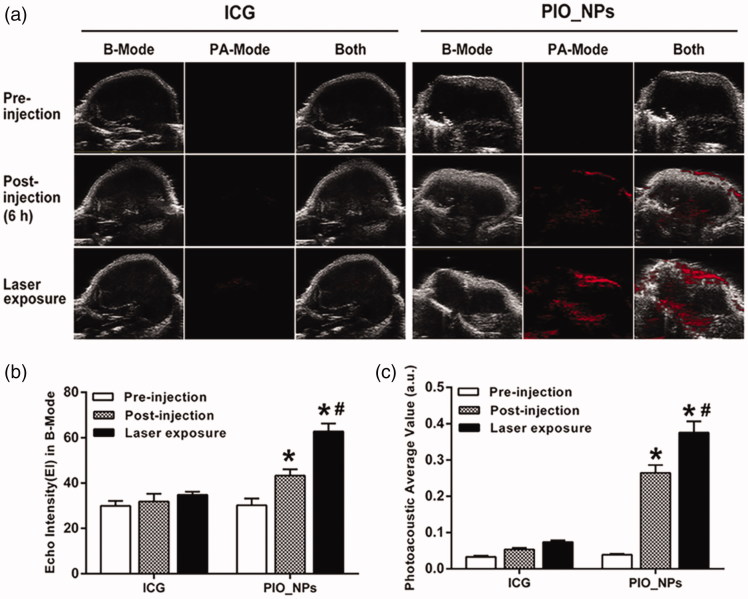
B-Mode and PA-Mode imaging of different groups before and after irradiation (a), echo intensity (b), and photoacoustic average value (c) were measured. Compared with pre-injection, echo intensity and PA value of PIO_NPs group increased, *p < .001. And EI and PA value were increased significantly after irradiation, #*p* < .05.

In order to investigate the time of PIO_NPs arriving and accumulating in tumor tissue, PA imaging was obtained at different times after PIO_NPs injection (Figure S6). PA signal was enhanced at the tumor site after 2–12 h of injection, and it was the strongest at 6 h, indicating that the PIO_NPs could accumulate in tumor tissue and enhance PA imaging. It also suggested that the L.U exposure should be performed after 6 h of PIO_NPs injection. Afterward, we compared the PA signal between free ICG and PIO_NPs groups (Figure 3). For PIO_NPs group, photoacoustic average value (a.u.) was increased from 0.26 ± 0.02 to 0.37 ± 0.03 due to laser irradiation. It may be related to the vaporization of PFP and thermal expansion of tissues (Wilson et al., 2012). However, there was no obvious PA signal for free ICG group. Because the clearance process of ICG was rapid (Wang et al., 2013; Wang et al., 2018), the majority of ICG was cleared out of body after 6 h.

### 3.4. Therapeutic effect *in vivo*

**Figure 4.**
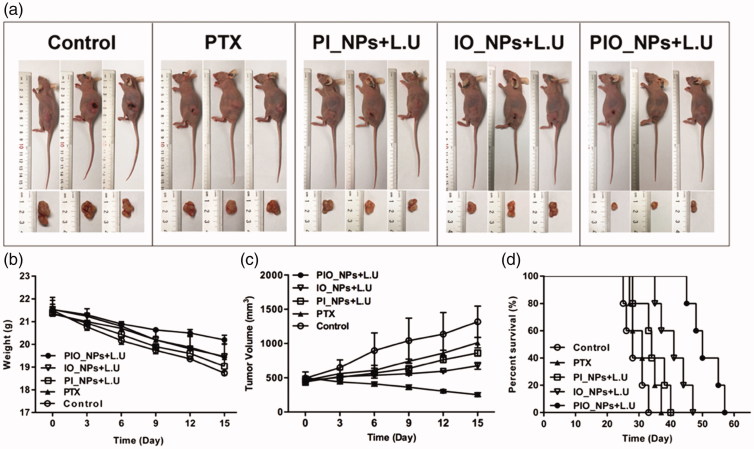
Growth inhibition of SKOV3 derived tumors in tumor xenograft models (a). During treatment, the body weight (b) and tumor volume (c) were recorded every 3 d. The tumors’ volume in experimental groups was significantly smaller compared to that in the saline control group. The mice in PIO_NPs + L.U group exhibited the slowest tumor growth rate. The survival curves of tumor-bearing mice treated in five groups (d). The median survival times of mice in PIO_NPs + L.U group was longer compared with other groups (*p* < .05).

During treatment, the tumors in the control group grew rapidly to ∼1300 mm^3^. When treated with PTX alone, the tumor growth was slightly inhibited (Figure 4(a,c)). Although PTX is a type of chemotherapeutic agent for ovarian cancer, it requires high dosage to reach anticancer effect. In our study, the amount of single PTX did not achieve a satisfying anticancer effect. In addition, the hypoxic environment in solid tumor negatively affected the effectiveness of chemotherapy (McEwan et al., 2015). For PI_NPs + L.U group, there was no significant difference in tumor volume compared with the PTX group. Without PFCs core, the stability of NPs may decrease and subsequently lead to a premature release of the encapsulated PTX (Rapoport, 2012). Tumor volume in IO_NPs + L.U group was not obviously increased. This may be due to the fact that IO_NPs can induce PSDT. Notably, the PIO_NPs + L.U group showed the optimal therapeutic effect with the tumor volume decreasing to about 250 mm^3^. These results indicated that PIO_NPs harbored the ability to combine chemotherapy and PSDT. Moreover, the oxygen released from PIO_NPs reduced hypoxia in tumor tissue (Liu et al., 2015) and improved the anti-tumor effects. During treatment, the average body weight (Figure 4(b)) of PIO_NPs + L.U group was decreased by nearly 1 g, but it was decreased by about 4 g in control group. After treatment, all remaining tumor-bearing mice were observed (Figure 4(d)). The corresponding median survival times in control, PTX, PI_NPs + L.U, IO_NPs + L.U and PIO_NPs + L.U group were 28, 31, 34, 41, and 50 d. Survival time of mice in PIO_NPs + L.U group was longer compared with other groups (*p* < .05). All of these results demonstrated that the PIO_NPs combined L.U enhanced the anti-tumor efficiency.

**Figure 5.**
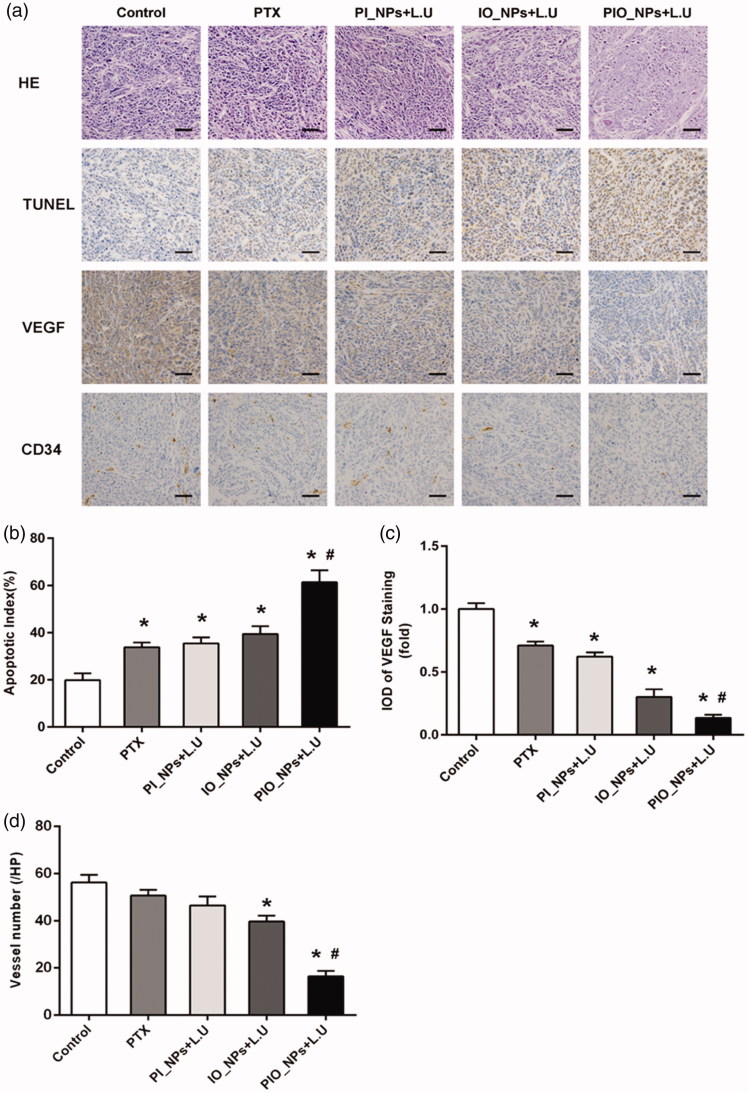
H&E staining, TUNEL assay, VEGF, and CD 34 immunohistochemical staining of tumor sections obtained 24 h after the last treatment from each group (a)scale bar is 50 μm. For TUNEL assay, cancer cell that was brown in nuclei were counted as the apoptotic cells. The apoptotic index(AI) in each group (b). The slide integral optical density (IOD) of VEGF protein in each group (c). CD34 immunohistochemical staining, the number of microvessels (brown) were counted. Quantitative analysis of microvessel density (MVD) in each group (d). Compared with control group, *p < .005; Compared with PIO_NPs + L.U group, #*p* < .05.

Furthermore, the H&E and immunohistochemistry (IHC) staining microscopic images of tumor sections were shown in Figure 5. In H&E staining images, there was obvious necrosis in PIO_NPs + L.U group, showing nuclear pyknosis, karyorrhexis, and karyolysis. However, there was no apparent necrosis in control or PTX group. In addition, tumor cell apoptosis after treatment was detected by TUNEL assay. Nuclei of apoptosis in tumor cells appeared as brown. For PIO_NPs + L.U group, the strongest positive staining was displayed among all groups. Other groups displayed weak or moderate positive staining, respectively. Quantitative analysis of apoptosis indexes (AI) also proved the consistent conclusion. According to Figure 5(b), the apoptosis indexes (AI) for treatment group PBS, PTX, PI_NPs + L.U and IO_NPs + L.U are 19.83 ± 2.89, 33.73 ± 2.02, 35.41 ± 2.54, and 39.43 ± 3.29%, respectively. Whereas this for PIO_NPs + L.U was 61.36 ± 5.08%, which was significantly higher compared with other treatment groups (*p* < .05). These results indicated that L.U mediated delivery of PIO_NPs significantly increased the tumor apoptosis efficiency.

As a key regulator of pathological angiogenesis, vascular endothelial growth factor (VEGF) contributes to growth and aggression of ovarian cancer (Masoumi Moghaddam et al., 2012). In our study, the expression of VEGF was detected by IHC staining (Figure 5), in which the positive cellular cytoplasm stained brown. The result showed that the expression of VEGF was the lowest in the PIO_NPs + L.U group. Then, IOD of VEGF positive staining was quantitatively analyzed (Figure 5(c)). The IOD in groups PTX, PI_NPs + L.U, and IO_NPs + L.U were 0.71 ± 0.02, 0.62 ± 0.01, and 0.30 ± 0.02 fold of the control group, respectively. There was a statistically significant difference between each treatment group and the control group (*p* < .05). Compared with PTX group, the VEGF expression was significantly inhibited in IO_NPs + L.U or PIO_NPs + L.U group, indicating that oxygen may be a relevant factor to reduce VEGF expression. The IOD of VEGF in PIO_NPs + L.U group was 0.13 ± 0.01 fold of the control group, which was the lowest among all groups. The low expression of VEGF demonstrated that angiogenesis in tumor can be effectively inhibited by the PIO_NPs combined L.U treatment. We also detected the expression of CD34 to determine the MVD in tumor tissue (Figure 5(d)). The CD34-MVD of the control, PTX, PI_NPs + L.U, and IO_NPs + L.U groups were 56.20 ± 3.27, 50.67 ± 2.42,46.50 ± 3.78, and 39.67 ± 2.42, respectively. For PIO_NPs + L.U group, CD34-MVD was 16.33 ± 2.34, which was significantly lower than other groups (*p* < .05). These results suggested that the PIO_NPs combined with L.U could suppress the formation of new vessels in tumor and further inhibit the growth of tumor.

**Figure 6.**
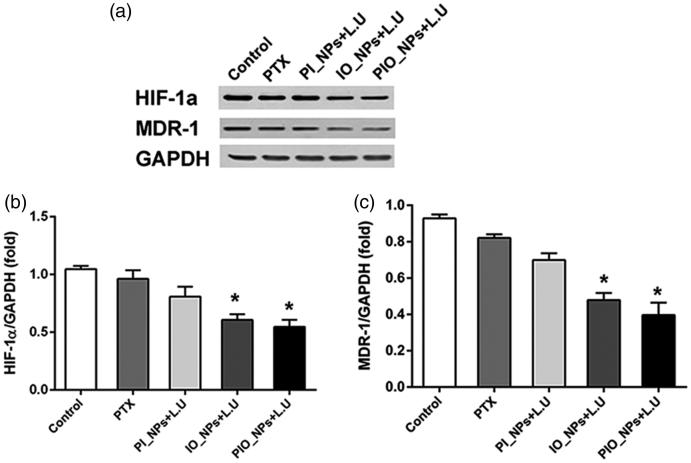
Expression of HIF-1a and MDR-1 in tumor tissue was detected by western blot analysis 24 h after treatment. Quantification of band intensity of HIF-1α expression relative to GAPDH was shown in columns (b). The intensity in control group to PIO_NPs + L.U group were 1.04 ± 0.03, 0.96 ± 0.07, 0.81 ± 0.09, 0.60 ± 0.05, 0.65 ± 0.2, and 0.54 ± 0.06, respectively. Compared with control group, *p < .005. Quantification of band intensity of MDR-1 expression relative to GAPDH was shown in columns (c). The intensity in control group to PIO_NPs + L.U group were 0.92 ± 0.02, 0.82 ± 0.01, 0.70 ± 0.03, 0.48 ± 0.04, and 0.40 ± 0.07, respectively.

The expression of HIF-1α and MDR-1 were detected by Western blot analysis (Figure 6). Compared with control group, the expression of HIF-1α and MDR-1 was significantly decreased in the IO_NPs + L.U group and the PIO_NPs + L.U group. However, there was no significant difference in other groups. These results proved that oxygen-carrying nanoparticles can improve hypoxia and reduce chemoresistance in tumor tissues (Liu et al., 2015; McEwan, et al., 2015; McEwan et al., 2016).

## 4. Conclusions

In summary, we constructed a type of phase-changeable nanoparticle that can carry oxygen and load ICG and PTX for ovarian cancer imaging and therapy. This nanoparticle preserved the optical properties of ICG and the chemotherapeutic effect of PTX, which also applied the phase-shift and carrying oxygen ability of PFP. And the PIO_NPs realized controlled release of PTX and generation of ROS by near-infrared laser and low-intensity ultrasound exposure. Based on the encapsulation of ICG and PFP, the PIO_NPs enhanced ultrasound and photoacoustic imaging. In addition, the accumulation of PIO_NPs in the tumor can be observed by PA imaging. These results suggested that the PIO_NPs might be a potential dual-mode imaging contrast agent, which can be used to monitor and guide treatment. The PIO_NPs combined with L.U exposure demonstrated significant anti-tumor efficacy against SKOV3 cell line and tumor. The anti-tumor mechanisms are considered in the following three aspects: (1) L.U-triggered PTX release; (2) ICG-based PSDT, with ROS producing; (3) oxygen transport to tumor site to enhance therapeutic effect and reduce hypoxia. Therefore, the PIO_NPs could be a promising drug delivery nanoplatform and imaging contrast agent, which might be used to realize integration of diagnosis and treatment for ovarian cancer.

## Supplementary Material

Supplementary Materials
