# Supplementary material for: Dual-mode imaging and therapeutic effects of drug-loaded phase-transition nanoparticles combined with near-infrared laser and low-intensity ultrasound on ovarian cancer
Source: Drug Deliv. 2018 Oct 20;25(1):1683–93. doi: 10.1080/10717544.2018.1507062 (PMC6201789; doi:10.1080/10717544.2018.1507062)
Supplement: Supplementary Materials [file IDRD_A_1507062_SM1015.docx]

***Supplementary***

**Dual-Mode imaging and therapeutic effects of drug-loaded phase-transition nanoparticles combined with near-infrared laser and low-intensity ultrasound on ovarian cancer**

Shuning Chen^1,3#^, Yujiao Liu^1#^, Shenyin Zhu^2^, Chunyan Chen^1^, Wan Xie^1^, Linlin Xiao^1^, Yi Zhu^1^, Lan Hao^3^, Zhigang Wang^3^, Jiangchuan Sun^1, *^，Shufang Chang^1, *^

*1Department of Obstetrics and Gynecology, The Second Affiliated Hospital of Chongqing Medical University, Chongqing 400010, China*

*2Department of Pharmacy, The First Affiliated Hospital of Chongqing Medical University, Chongqing 400016, China*

*3Institute of Ultrasound Imaging, Second Affiliated Hospital of Chongqing Medical University, Chongqing 400010, China*

*#These authors contributed equally to this work.*

*Correspondence to: Shufang Chang, MD, Professor of Obstetrics and Gynecology. Department of Obstetrics and Gynecology, The Second Affiliated Hospital of Chongqing Medical University, 74 Linjian Road, Yuzhong District, Chongqing 400010, China. Tel: +86 23 6369 3279, Fax: +86 23 6510 4238, Email:* [*shfch2005@163.com*](mailto:shfch2005@163.com) *or Jiangchuan Sun, Professor of Obstetrics and Gynecology. Department of Obstetrics and Gynecology, The Second Affiliated Hospital of Chongqing Medical University, 74 Linjian Road, Yuzhong District, Chongqing 400010, China. Tel: +86 23 6369 3483, Fax: +86 23 6510 4238, Email:* [*sunjiangchuan@126.com*](mailto:sunjiangchuan@126.com)

**Supplementary Methods**

1. ***Dual-model imaging in vitro***

B-mode, contrast mode and PA-mode imaging of the PIO_NPs were investigated. PBS for control, free ICG and O_NPs were also studied as comparison. We used an agar gel phantom (2% agar w/v in distilled water) to evaluate the function of PIO_NPs as a contrast agent for dual-mode imaging. A pipette tip was inserted into gel to create a hole into which the NPs or free ICG solution was deposited. During each agent exposed to laser-irradiation (808nm diode laser, Mid-River Ltd., Xi’an, China) using 1.5W/cm2 for 2 min, B-mode and contrast mode images were captured by ultrasonic diagnostic instrument (MyLab 90, Esaote, Italy) using a liner probe (5-12MHz). Then, those images were analyzed using DFY (invented by the Institution of Ultrasound Imaging of Chongqing Medical University) to measure the Echo Intensity (EI) in B-mode and contrast mode separately. PA-mode images of the samples were obtained and analyzed using the VEVO LASR PA imaging system (VIVO 2100, FUJIFILM Visual Sonic, Inc., USA) by following method described above.

1. ***In vitro MTT assay***

The SKOV3 cells were seed into 48-well plate (1.4×10^5^/well) in 250μL of medium and cultured for 24h. To choose the appropriate treatment concentration, the medium was changed to different concentration PTX or PIO_NPs. After 4h incubation, sterile PBS washed cells thrice and each well add new complete medium. Then the treated cells were irradiated with 1.5W/cm2 808nm for 2min and exposed to 1.0 W/cm^2^ low-intensity for 1min (The role of laser and ultrasound is abbreviated as L.U). Afterwards cells were incubated for another 24h, the cell viability was quantified by MTT assay. We also use MTT assay to explore the effect of different treatments. After cell planted 24h, the medium was removed and replaced by 250μL culture media containing equivalent concentrations of free PTX, IO_NPs, PI_NPs and PIO_NPs (final ICG concentration 3.46μg/mL, PTX 6.90μg/mL). The rest of the procedure is the same as above.

**Supplementary Figures and Tables**

|  | Diameter | Zeta potential | Polydispersity | EE of PTX | EE of ICG | LE of PTX | LE of ICG |
| --- | --- | --- | --- | --- | --- | --- | --- |
|  | (nm) | (mV) |  | (%) | (%) | (%) | (%) |
| PIO_NPs | 186.4±1.9 | -19.43±0.55 | 0.112±0.019 | 64.29±2.76 | 57.27±0.77 | 3.64±0.16 | 1.73±0.15 |
| IO_NPs | 191.0±5.1 | -20.40±1.55 | 0.092±0.003 | - | 52.45±1.34 | - | 1.30±0.30 |
| PI_NPs | 214.3±6.3 | -13.67±2.65 | 0.162±0.042 | 57.59±3.36 | 51.44±2.26 | 3.46±0.21 | 1.11±0.05 |

**Table S1**. Size and zeta potential of PIO_NPs, IO_NPs, PI_NPs. LE and EE of PTX or ICG for different nanoparticles. Values are mean± SD (n=3).

**Notes:** EE, drug encapsulation efficiency; LE, drug loading efficiency.


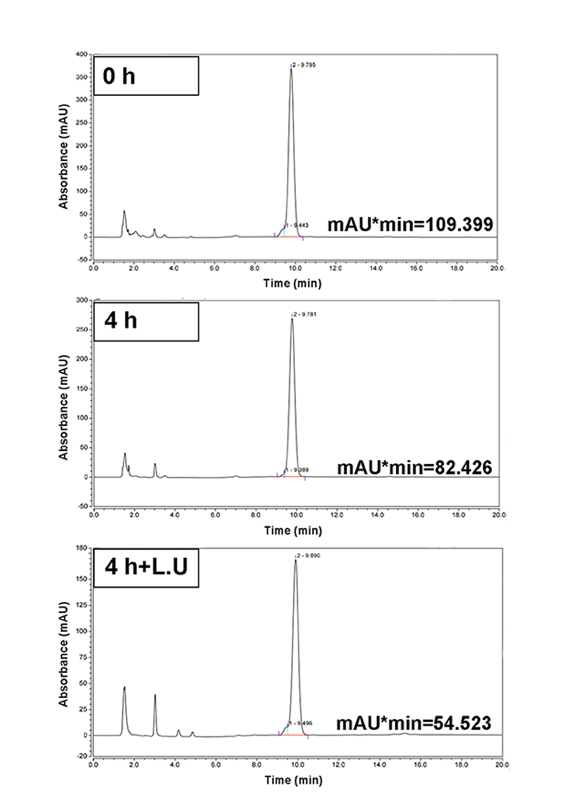


**Figure S1**. The PTX contained in PIO_NPs were detected by HPLC at different times at 37°C and pH 7.4. The release of PTX was increased after laser and ultrasound exposure at 4 h.


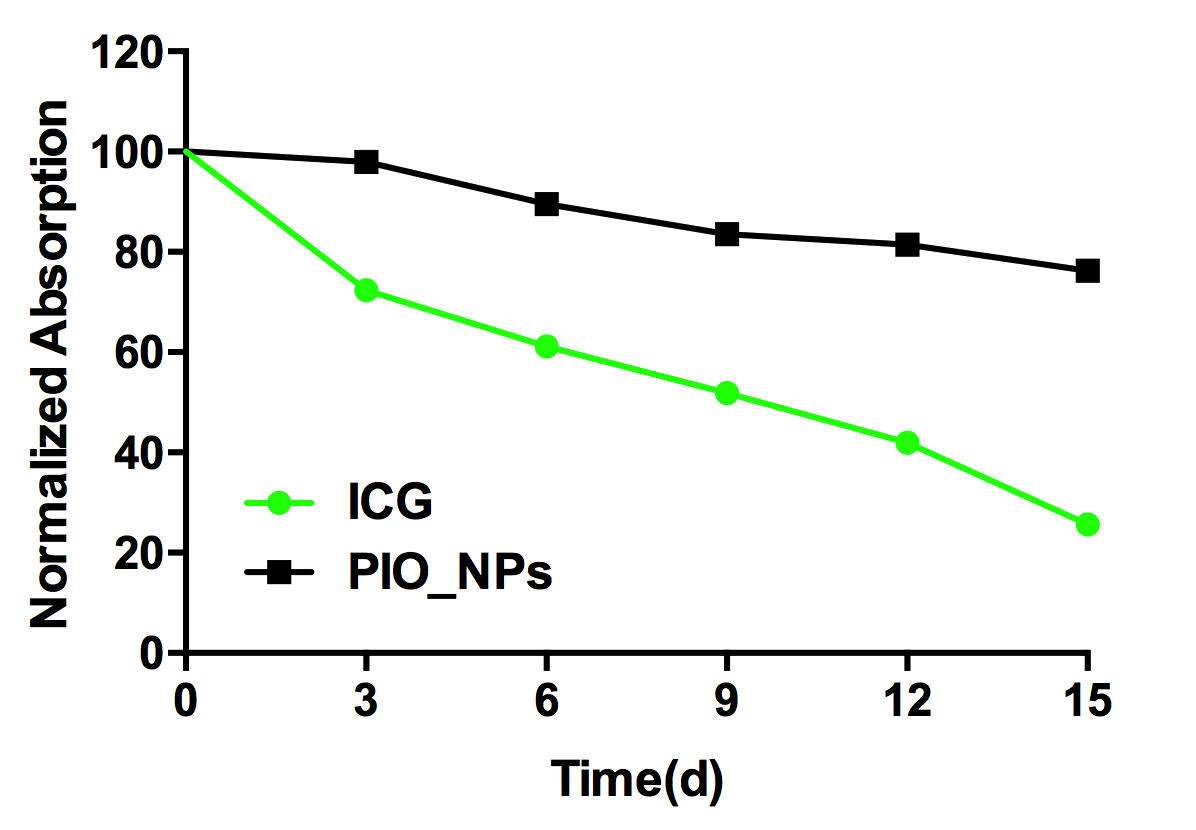


**Figure S2.** Normalized absorption of PIO_NPs and free ICG in 10% fetal bovine serum every three days. After 15-day observation, PIO_NPs decreased the intensity of absorption by about 25%, while free ICG was decreased nearly 80%.


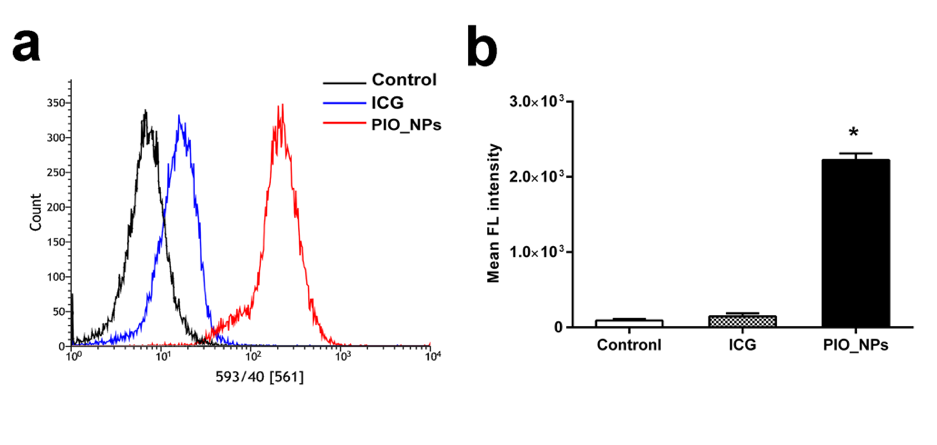


**Figure S3.** Flow analysis of mean fluorescence intensity in SKOV3 cells incubated with media (black line), ICG (blue line) and PIO_NPs (red line) (**a**). Mean fluorescence intensity of different groups (**b**). The PIO_NPs group was significantly higher than others, *P< 0.005.


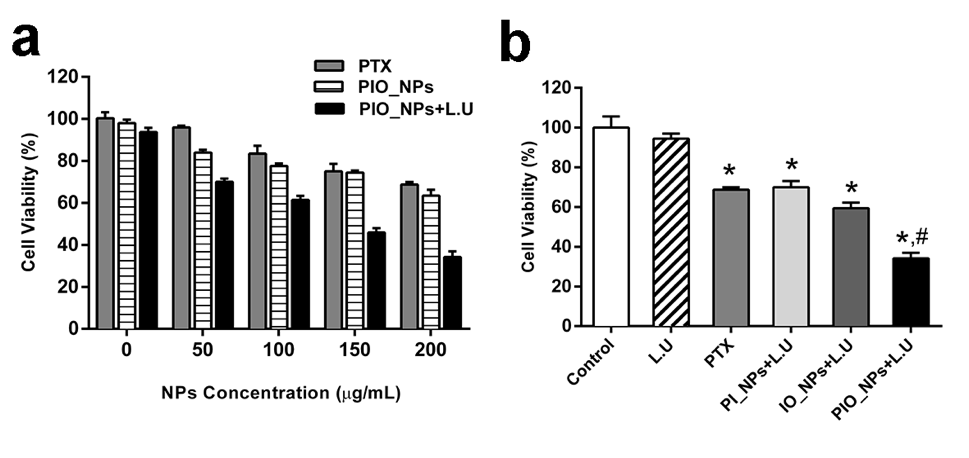


**Figure S4.** Cytotoxicity of different concentration of PTX, PIO_NPs and PIO_NPs plus laser and ultrasound (**a**). Cytotoxicity of different treatment groups (**b**). Compared with control, *P<0.005; PIO_NPs+L.U group compared with other groups, #P<0.005.


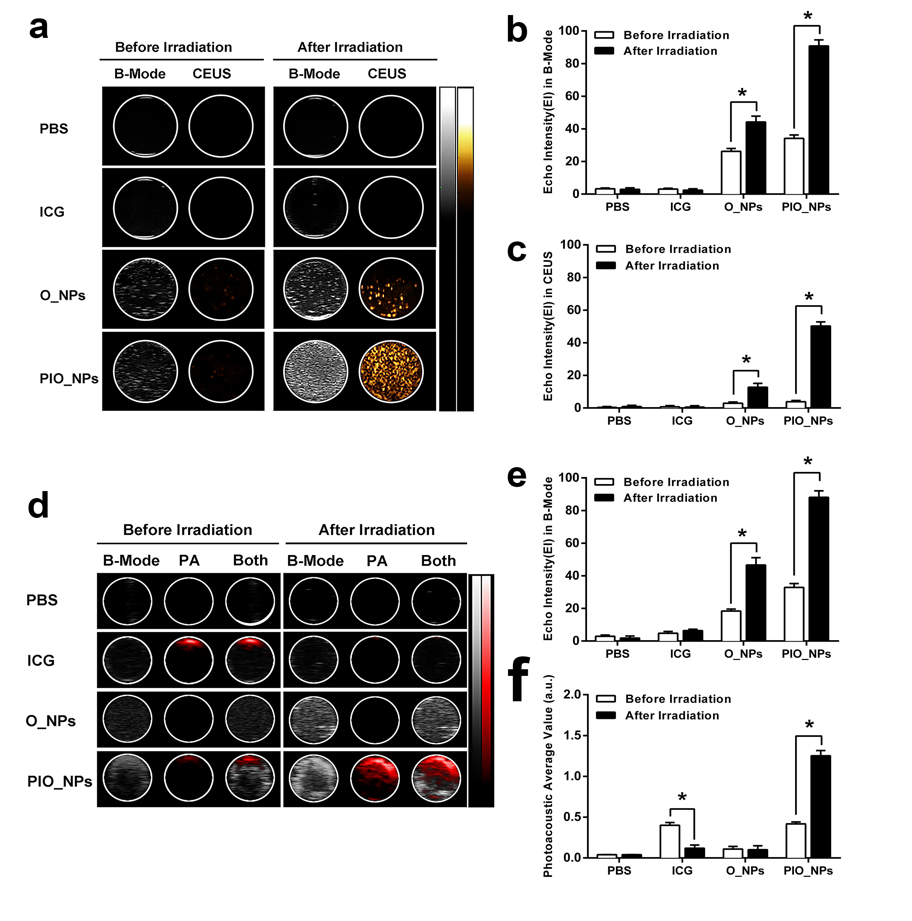


**Figure S5.** Dual-mode imaging in vitro. B-mode and contrast mode (CEUS) imaging of different groups before and after irradiation(**a**). Echo intensity (EI) in B-mode (**b**) and in CEUS (c) of different groups. After irradiation, Echo intensity of O_NPs and PIO_NPs increase significantly, * P<0.005. B-Mode and PA-Mode imaging of different groups before and after irradiation (**d**). Echo intensity (**e**) and Photoacoustic average value (**f**) were measured. PA value of PIO_NPs increased after irradiation, but it decreased in ICG group significantly. All values are mean± SD (n=3), * P<0.005.


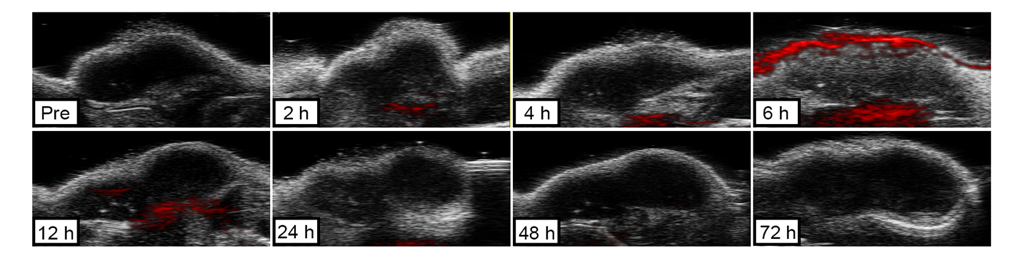


**Figure S6.** PA imaging in different time after injected PIO_NPs. PA signals can be acquired within 2 to 12 hours, with the strongest at 6 h.
